# Decreased reward circuit connectivity during reward anticipation in major depression

**DOI:** 10.1016/j.nicl.2022.103226

**Published:** 2022-10-10

**Authors:** Hanneke Geugies, Nynke A. Groenewold, Maaike Meurs, Bennard Doornbos, Jessica M. de Klerk-Sluis, Philip van Eijndhoven, Annelieke M. Roest, Henricus G. Ruhé

**Affiliations:** a Martini Ziekenhuis, Groningen, the Netherlands; b Interdisciplinary Centre Psychopathology and Emotion regulation (ICPE), UMCG, Groningen, the Netherlands; c Department of Psychiatry and Mental Health, Neuroscience Institute, University of Cape Town, Cape Town, South Africa; d IJ-lab Therapieland, Amsterdam, the Netherlands; e Lentis, Groningen, the Netherlands; f Department of Psychiatry, Radboudumc, Nijmegen, the Netherlands; g Donders Institute for Brain, Cognition and Behavior, Radboud University, Nijmegen, the Netherlands; h Developmental Psychology, University of Groningen, Groningen, the Netherlands

## Abstract

An important symptom of major depressive disorder (MDD) is the inability to experience pleasure, possibly due to a dysfunction of the reward system. Despite promising insights regarding impaired reward-related processing in MDD, circuit-level abnormalities remain largely unexplored. Furthermore, whereas studies contrasting experimental conditions from incentive tasks have revealed important information about reward processing, temporal difference modeling of reward-related prediction error (PE) signals might give a more accurate representation of the reward system. We used a monetary incentive delay task during functional MRI scanning to explore PE-related striatal and ventral tegmental area (VTA) activation in response to anticipation and delivery of monetary rewards in 24 individuals with MDD versus 24 healthy controls (HCs). Furthermore, we investigated group differences in temporal difference related connectivity with a generalized psychophysiological interaction (gPPI) analysis with the VTA, ventral striatum (VS) and dorsal striatum (DS) as seeds during reward versus neutral, both in anticipation and delivery. Relative to HCs, MDD patients displayed a trend-level (p = 0.052) decrease in temporal difference-related activation in the VS during reward anticipation and delivery combined. Moreover, gPPI analyses revealed that during reward anticipation, MDD patients exhibited decreased functional connectivity between the VS and anterior cingulate cortex / medial prefrontal cortex, anterior cingulate gyrus, angular/middle orbital gyrus, left insula, superior/middle frontal gyrus (SFG/MFG) and precuneus/superior occipital gyrus/cerebellum compared to HC. Moreover, MDD patients showed decreased functional connectivity between the VTA and left insula compared to HC during reward anticipation. Exploratory analysis separating medication free patients from patients using antidepressant revealed that these decreased functional connectivity patterns were mainly apparent in the MDD group that used antidepressants. These results suggest that MDD is characterized by alterations in reward circuit connectivity rather than isolated activation impairments. These findings represent an important extension of the existing literature since improved understanding of neural pathways underlying depression-related reward dysfunctions, may help currently unmet diagnostic and therapeutic efforts.

## Highlights

• MDD patients showed decreased temporal difference-related ventral striatum activation during reward anticipation and delivery vs. controls.
• In gPPI analyses, during reward anticipation, MDD patients showed decreased functional connectivity in the reward-network.
• Exploratory analyses showed functional connectivity patterns were mainly apparent in the MDD group that used antidepressants.

## 1 Introduction

One of the core characteristics of major depressive disorder (MDD) is anhedonia, the inability to experience pleasure. Anhedonia affects approximately 37 % of individuals diagnosed with MDD (Pelizza and Ferrari, 2009). A dysfunction of the reward system is thought to comprise the neural basis of anhedonia (Der-Avakian and Markou, 2012, Pizzagalli, 2014, Treadway and Zald, 2011, Whitton et al., 2015). The presence of anhedonia has been found to predict poor treatment response in MDD patients (Spijker et al., 2001, Uher et al., 2012), and impairments in reward-relates processes appear to be insufficiently addressed by current treatments (Calabrese et al., 2014).

In recent years, a significant number of studies have sought to identify the neural correlates of reward-related processes (Berridge et al., 2009, Der-Avakian and Markou, 2012, Pujara and Koenigs, 2014, Whitton et al., 2015). Most notably, the dorsal striatum (DS), i.e., the caudate, the ventral striatum (VS), i.e., the nucleus accumbens, and the ventral tegmental area (VTA) have been found to play an important role in reward processes (Fareri et al., 2008, O'Doherty, 2004, Russo and Nestler, 2013). More specifically, depressed individuals showed decreased striatal activity (ventral and dorsal) in response to reward anticipation (Pizzagalli et al., 2009, Smoski et al., 2009, Zhang et al., 2013) and reward delivery (Admon et al., 2015b, Smoski et al., 2009, Zhang et al., 2013). Furthermore, increased activation was observed in frontal regions including the middle frontal gyrus and the anterior cingulate cortex (ACC) in MDD patients during reward anticipation (Zhang et al., 2013).

Neural reward processing has been related to phasic firing of dopaminergic neurons (Schultz, 1998, Tobler et al., 2005) and is often studied with the Monetary Incentive Delay (MID) task. The MID gives the participant an opportunity to either win or lose rewards based on how fast they respond to a target, and covers two phases of reward processing: anticipation versus delivery (Knutson et al., 2001). In incentive trials, dopamine activity is dependent on the combination of reward anticipation (expectancy) and the subsequent delivery (i.e., consumption or outcome) of the reward. When a reward is anticipated but omitted, there is a decrease in dopaminergic firing (referred to as a negative prediction error (PE)) whereas a phasic burst of dopamine (i.e., positive PE) is observed when the reward delivery is better than expected (Schultz, 1998). Positive and negative PEs can be used as parametric modulators in order to reflect the magnitude of dopaminergic activation. PEs have been predominantly used in fMRI related reinforcement learning models in order to capture reward learning signals (Dombrovski et al., 2015, Geugies et al., 2019, Gradin et al., 2011, Kumar et al., 2008, Rothkirch et al., 2017). However, PEs also exist in incentive fMRI tasks without an explicit learning compound like (card-) guessing tasks or the MID task (Chase et al., 2013, Staudinger et al., 2009, Ubl et al., 2015, Yacubian et al., 2006, Cao et al., 2019), although PEs here are often not distinctively examined.

Whereas studies contrasting experimental conditions from incentive tasks have revealed important information about the neural correlates of reward processing, temporal difference modeling of reward-related PE-signals might give a more accurate representation of the reward system (Staudinger et al., 2009). So far, only few studies investigated reward-related PE signaling in depression. Reinforcement learning studies found increased activation of the VTA (Geugies et al., 2019, Kumar et al., 2008) and decreased VS (Gradin et al., 2011, Kumar et al., 2008) and DS (Gradin et al., 2011) activity in (remitted) MDD. Reward expectancy studies revealed reduced frontal and striatal activity during anticipation of gain (Chase et al., 2013, Ubl et al., 2015) and losses (Ubl et al., 2015) in MDD. Moreover, these altered reward-related processes in depressed individuals seems to be substantially associated with anhedonia. Several studies report a negative correlation between anhedonia and basic reward activity in the VS (Der-Avakian and Markou, 2012), as well as temporal difference-related VS activity (Rothkirch et al., 2017), during reward processing in MDD. However, one recent study found that higher anhedonia was associated with higher VS activity during anticipation in MDD (Ubl et al., 2015).

Despite these promising insights regarding neural correlates, there is evidence that MDD is associated with alterations in connectivity between components of the reward circuitry in addition to dysfunction of individual brain areas (Admon et al., 2015b). Admon and colleagues found decreased connectivity between the caudate (i.e. DS) and the dorsal ACC in response to monetary loss outcome and increased connectivity between these two regions in response to monetary gain outcome in MDD patients (Admon et al., 2015b). In line with this finding, Dombrovski and colleagues demonstrated disrupted connectivity between the DS and prefrontal cortex during probabilistic reversal learning in patients with late-life depression (Dombrovski et al., 2015). Despite these interesting findings, it remains largely unexplored if alterations in connectivity between other elements of the reward circuitry besides the DS (i.e. the VS and VTA), exist and whether these alterations can be linked to depression.

Therefore, this study aimed to (I) investigate PE-related striatal and VTA activation in MDD in response to anticipation and delivery of monetary rewards (providing a more accurate representation of the reward system), and explore the association with anhedonia. Furthermore, we (II) also wanted to investigate, with an exploratory approach, whether MDD is characterized by alterations in connectivity within the reward circuitry, by looking at abnormal striatal (VS and DS) and VTA connectivity in response to rewards. In line with the literature, we expected reduced PE-related activity in MDD patients compared to healthy controls (HC) in the VS (Kumar et al., 2008, Pizzagalli et al., 2009) and DS (Admon et al., 2015b, Pizzagalli et al., 2009) and increased activation of the VTA (Kumar et al., 2008) during both reward anticipation and outcome. In addition, we expected a negative correlation between reward activity and anhedonia severity during reward processing (Der-Avakian and Markou, 2012, Rothkirch et al., 2017). Moreover, decreased reward-circuitry connectivity in MDD patients compared to HC was expected for the VTA, the VS and the DS (Admon et al., 2015b).

## 2 Material and methods

### 2.1 Participants

Data was derived from the Depression In the Picture (DIP) neuroimaging study conducted at the University Medical Center Groningen investigating the neural correlates of depression. Permission for the study was obtained from the local ethics committee and written informed consent obtained from all participants. Twenty-four MDD patients were recruited through specialized mental health care institutions and advertisements at the participating institutions and satisfied the following criteria: (1) presence of at least mild depressive symptoms defined as a Beck Depression Inventory (BDI-II) (Beck et al., 1996) score > 13 at screening, (2) current depressive disorder diagnosis according to the MiniScan (Nienhuis et al., 2010), administered by trained postgraduate students, and 3) age ≥ 18 years. Twenty-four age- and sex-matched HC were recruited by means of advertisements at public places and in local newspapers. Inclusion criterion for HC was a BDI-II < 9 and HCs were excluded if there was a personal history of psychiatric disorders. General exclusion criteria for both groups were: (1) a current or lifetime diagnosis of drug dependence, excluding nicotine dependence or history of alcohol dependence/abuse, (2) current neurological problems that may interfere with task performance, (3) inadequate comprehension of the Dutch language, (4) MRI contraindications such as metal implants, (5) presence of any cardiovascular disease. Exclusion criteria specific for MDD patients were: (1) presence of current or lifetime psychiatric disorders other than MDD or anxiety disorders, (2) concrete suicidal plans, (3) psychotropic medication use other than SSRI/SNRI/TCA or infrequent benzodiazepine use.

### 2.2 Task

After a short practice run before scanning, participants performed a monetary incentive delay (MID) task to asses reward processing. The task was a shortened version of the task design previously described by Pizzagalli et al. (2009). The task consisted of 4 blocks of 13 trials with a total of 20 reward trials, 20 neutral trials, and 12 loss trials. Each trial consisted of the presentation of a cue (+€ / ±€ / −€ indicating a reward, neutral or loss trial), a target presentation (blue square), and reward feedback (i.e., +€1.85). Cues and feedback were presented for 1.5 s and the target was presented for a fixed duration of 0.5 s. Monetary outcomes trials varied for successful reward (+€1.75, +€1.85, +€1.95 and +€2.05) and loss (−€1.60, −€1.70 and −€1.80), but were fixed at +€0.00 for non-reward and neutral trials. We used fixed reward success rates because monetary outcomes were determined by task order (not response time). The 80 % reward success rate was chosen to ensure that task activation would be detectable for both anticipation and outcome phases of the task. At very high reward success rates, task activation will be much stronger in the anticipation phase and sensitivity is lost in the outcome phase. However, when reward success rates are at chance level, task activation is expected to be much stronger in the outcome phase and by limited strength of the learned association between cue and outcome, sensitivity is lost in the anticipation phase (Schultz, 2007). Unsuccessful reward trials ensured sufficient variability in reward prediction errors. The inter-stimulus interval varied between trials (inter-stimulus interval between cue and target: 3.5 s – 9.5 s; inter-stimulus interval between target and feedback: 2.5 s – 8.5 s) to prevent expectancy effects, as was the duration of the fixation cross presented between trials (3 s – 7 s). Stimuli were presented in E-prime 2 (Psychology Software Tools, Pittsburgh, PA). Given our aims, neural correlates of loss trials were not examined, but maintained for comparability with previous MID studies and to prevent participants from associating neutral trials with a loss experience. Participants were instructed to press the button on an MRI-compatible button box as quickly as possible after target presentation on each trial, in order to maximize their chances of obtaining a reward. If a participant neglected to press the button, no reward could be obtained for that trial. Reward success rates were fixed at 80 % to ensure a total obtained reward of €10 per participant. This reward was added to the financial compensation for participation, to increase motivation of the participants.

### 2.3 Data acquisition

Functional images were acquired on a Philips 3-Tesla MR-scanner equipped with a 32-channel SENSE head coil. T2*-weighted images were acquired with the following parameters: 425 whole-brain volumes; repetition time 2000 ms; echo time 20 ms; flip angle 70°; 37 axial slices; no slice gap; 64 × 61 matrix; voxel size 3.5 × 3.5 × 3.5 mm; field of view (FOV) 224 × 129.5 × 224 mm. High resolution T1-weighted anatomical images were acquired with the following parameters: repetition time 9 ms; echo time 3.6 ms; 170 sagittal slices; 256 × 231 matrix; voxel size 1 × 1 × 1 mm.

### 2.4 Temporal difference learning model

In order to parametrically modulate fMRI signals, PEs after (repeated) rewards and during (unexpected) non-rewards were computed for the time series of stimuli. Unexpected non-rewards occurred when the button was pressed on time but no reward was obtained. The calculation of temporal difference PEs for all trials was derived from Staudinger and colleagues (Staudinger et al., 2009). This model defines a reward expectation EV that was defined as:

EV=m×p

where *m* is corresponding to the expected gain and *p* is the gain probability. As expected gain we chose average win and loss values from the practice run. The gain probability was set to 0.8 as 80 % of the reward trials resulted in an actual win and the other 20 % in an omission.

The PE was defined as:

PE=R-EV

where *R* is corresponding to the amount of reward that was actually received.

### 2.5 Analysis of sample characteristics

Sample characteristics and behavioral data was analyzed in SPSS package v22.0 (SPSS Inc., USA). We used independent samples t-tests, χ2-tests and non-parametric Mann-Whitney *U* test to compare demographic and clinical variables between MDD patients and the HC group.

### 2.6 Analysis of behavioral data

For anhedonia scores, we used a subscale measurement of the Beck Depression Inventory (loss of pleasure, interest, energy and libido; (Pizzagalli et al., 2009)). We used repeated measures analysis of variance to examine main effects of group (MDD and control) and condition (reward and neutral) and a group × condition interaction with reaction times as dependent variable.

### 2.7 Imaging data

Pre-processing and analysis were performed using SPM12 (https://www.fil.ion.ucl.ac.uk/spm) implemented in Matlab R2013a (The MathWorks Inc., Natick, MA). First the PAR/REC files were converted to NIfTI format. Both structural and functional images were reoriented in AC-PC alignment. Functional images were realigned. To detect possible motion artefacts, framewise displacement (FD) was calculated (Power et al., 2012). Motion was deemed excessive when FD > 0.9 for a certain volume (Siegel et al., 2014). However, the number of volumes with excessive motion was minimal (<10 %) for all participants. Median FD was 0.133 (IQR 0.036) for MDD patients and 0.127 (IQR: 0.039) for HC. We observed no significant difference in FD between the MDD patients and HC group (p = 0.224). Functional images were co-registered to the structural T1 images. All images were spatially normalized to Montreal Neurological Institute (MNI) space. Finally, all images were smoothed using an 8 mm Full Width Half Maximum Gaussian kernel.

### 2.8 Temporal difference-related activity

For each participant, first-level hemodynamic responses for the different conditions were modelled with general linear models. Reward anticipation, reward delivery, neutral anticipation, neutral delivery, loss anticipation and loss delivery were defined as regressors. Onset times were for the anticipation: start of cue presentation and for delivery: start of feedback. Durations for all regressors were 0.75 s. E-prime log files were used to extract onset times and durations. In separate GLMs, prediction errors were entered into the model as parametric modulators. The parametric modulation regressor was mean-corrected by SPM to be orthogonal to the main outome regressor (Staudinger et al., 2009). Low frequency noise was removed via a high pass filter (128 s). Furthermore, realignment parameters, their first derivatives and FD calculations were added to the model to address residual movement not corrected by realignment. For all participants, separate first-level contrasts for the total temporal difference-related activation (RewardAnticipation + RewardDelivery > NeutralAnticipation + NeutralDelivery) and for reward anticipation (RewardAnticipation > NeutralAnticipation) and reward delivery (RewardDelivery > NeutralDelivery) were defined and taken to second level.

A priori regions of interest (ROI) were the striatum (caudate and nucleus accumbens) and VTA. ROI selection was based on the Reinforcement Learning Atlas (Pauli et al., 2018). Accordingly, the boundaries of the Caudate (dorsal striatum) are clear with its tail traveling caudally and ventrally around the lateral ventricle. Exception is its ventral boundary with the nucleus accumbens (ventral striatum). We used the definition that the caudal limit of the NAC coincides with the appearance of the anterior commissure in coronal sections. The VTA lies ventral to the raphe nuclei (RN) at the ventromedial limit of the parabrachial pigmented nucleus (PBP) in coronal sections. Rostrocaudally, the VTA extends from the approximate rostrocaudal midpoint of the RN to just beyond the caudal limit of the RN (coronal sections). The boundary with the RN is a well defined and explicit, but the transition from PBP to VTA are more implicit (Pauli et al., 2018).

At second-level, we used a one sample *t*-test to investigate main effects of task (RewardAnticipation + RewardDelivery > Neutral contrast). Main effect images were thresholded at *P* < 0.001 uncorrected. We used independent two-sample t-tests to determine group differences. As we had clear *a priori* regions of interest, a small volume correction (SVC) was applied with significance defined as *P* < 0.05 FWE corrected.

### 2.9 Generalized psycho-physiological interaction (gPPI) analysis

We investigated group differences in temporal difference-related connectivity during the reward task with a generalized psychophysiological interaction (gPPI) analysis (McLaren et al., 2012) with VTA, ventral striatum and dorsal striatum as seeds during reward versus neutral, both in anticipation and delivery. The seeds were extracted from the Reinforcement Learning Atlas (Pauli et al., 2018) and were resliced to match the dimensions of the functional data. On first level, separate gPPI models for each seed were estimated for each participant. Each first level model contained regressors for the task conditions, one regressor for the seed, and regressors for the seed × condition interaction. Furthermore, realignment parameters, their first derivatives and FD calculations were added to the model to address residual movement not corrected by realignment. Effects for the obtained interaction variable were convolved using a canonical hemodynamic response function (HRF). For all participants, first-level contrasts for reward anticipation (RewardAnticipation > NeutralAnticipation) and reward delivery (RewardDelivery > NeutralDelivery) separate were defined and taken to second level (so no contrast with TD related activity was taken). On second level, we used independent two-sample t-tests to determine group differences. An initial threshold was set to *P* < 0.001 uncorrected (voxel level), where group differences were defined significant at *P* < 0.017 (Bonferroni correction *P* 0.05/3 ROIs), FWE cluster-level corrected.

In order to interpret temporal difference-related activation and connectivity findings, we also investigated correlations with anhedonia with separate multiple regression analyses with temporal difference-related activation signal and connectivity findings respectively as the dependent variable, while anhedonia scores, group and the group*anhedonia interaction were examined.

### 2.10 Exploratory analysis: Effect of medication

Because of two recent *meta*-analyses that indicate that some types of antidepressants may have a small positive effect on cognitive functioning (Keefe et al., 2014, Rosenblat et al., 2015), we chose to do an exploratory analysis by splitting up the patient group into a medication free group (MDDmed-, N = 14) and an antidepressant using MDD group (MDDmed+, N = 10) in order to rule out any medication effects on the results.

## 3 Results

### 3.1 Sample characteristics

No significant differences were observed between MDD patients and HC (Table 1). The exploratory analysis with three groups (HC vs MDD with/without medication) also revealed no significant differences between groups (Table 1).

**Table 1:** Demographic and Clinical Characteristics.

				MDD		HC vs. MDD all		HC vs. MDD_med+_, MDD_med-_	
		Healthy controls (N = 24)	MDD (all) (N = 24)	med+ (N = 10)	med- (N = 14)	Test-statistic	*p*	Test-statistic	*p*
Age (years)	Mean (range)	44 (24-67)	44 (23-69)	45 (30-66)	44 (23-69)	*t*(46) = -0.11	0.91	F(2,45) = 0.04	0.97
Sex	Male/Female	7/17	6/18	5/5	1/13	*X*^2^(1) = 0.11	0.75	*X*^2^(2) = 5.53	0.06
Education levels^a^	N (1/2/3/4/5/6/7)	0/1/0/1/6/9/7	0/0/0/1/7/8/8	0/0/0/1/3/2/4	0/0/0/0/4/6/4	*X*^2^(4) = 1.20	0.88	*X*^2^(8) = 3.71	0.88
BDI-II at MRI^b^	Median (IQR)	1 (0-3)	27.5 (16-31.75)	17.5 (12.5-28)	28.5 (22-33)	*U* = 0	< 0.001	*t*(22) = -1.89	0.07^d^
Anhedonia MRI^c^	Median (IQR)	0 (0-0)	3 (2-3)	2.5 (1.75-3.25)	3 (1.75-3)	*U* = 30.5	< 0.001	*U* = 67	0.89^d^
Age of onset MDD	Mean (range)	-	25 (8-65)	23 (16-32)	27 (8-65)	*-*	-	*t*(18) = 0.83	0.42^d^
Singe/Recurrent Episodes	N (single/recurrent/NS)	-	6/16/2	4/6/0	2/10/2	*-*	-	*X*^2^(2) = 3.09	0.21^d^
Comorbid anxiety	N (GAD/SAD/AG/none)	-	4/1/1/18	1/1/1/7	3/0/0/11	*-*	-	*X*^2^(3) = 3.31	0.35^d^
AD use	N (SSRI/SNRI/TCA)	-	6/2/2	6/2/2	-	*-*	-	*-*	-

MDD = major depressive disorder, ^a^Level of educational attainment (Verhage and van der Werff, 1964). Levels range from 1 to 7 (1 = primary school not finished, 7 = preuniversity/university degree), ^b^Beck Depression Inventory (BDI-II) total scores, ^c^Beck Depression Inventory (BDI-II) anhedonia-subscores, ^d^MDD med + versus MDD med-, IQR = Inter-quartile range, NS = not specified, GAD = generalized anxiety disorder, SAD = social anxiety disorder, AG = agoraphobia, AD = antidepressant.

### 3.2 Behavioral results

**Fig. 1:**
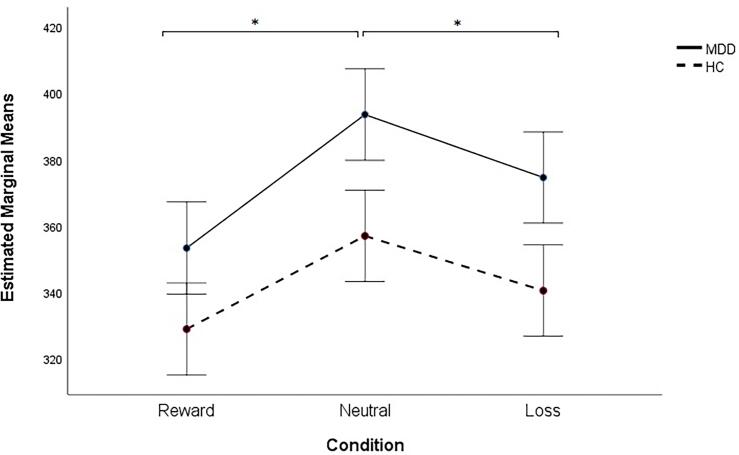
Reaction times for different conditions. Error bars refer to standard error of the mean. *P < 0.020.

We observed no significant differences in reaction times between the two groups (MDD versus HC) and observed no significant group × condition interaction (Fig. 1). There was a main effect of condition (F(2,92) = 10.79, *P* < 0.001). Post-hoc least significant difference (LSD) comparisons revealed that all participants reacted significantly faster to reward trials and to loss trials compared to neutral trials (P = 0.000 and P = 0.019, respectively).

### 3.3 Main effect of task

**Fig. 2:**
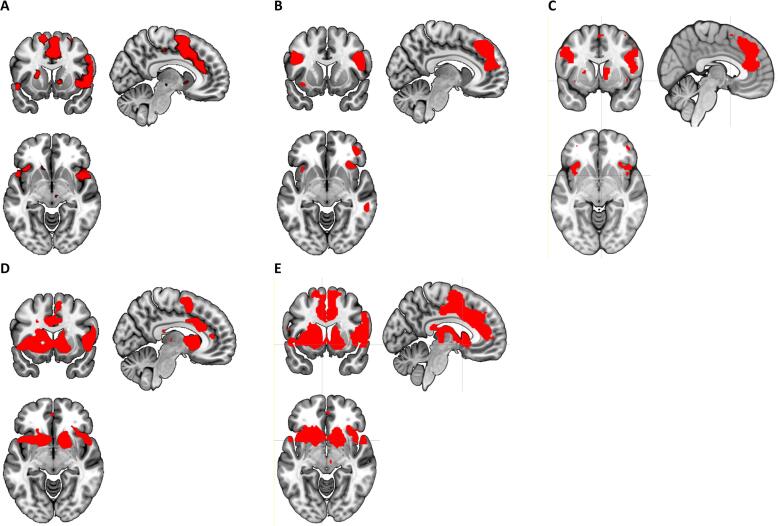
Main effect of task (>neutral) at P < 0.0001 uncorrected. A) task effect Reward Anticipation. B) task effect Reward Delivery. C) task effect Reward Anticipation + Reward Delivery. D) Reward Delivery with TD modulation. E) Reward Anticipation + Reward Delivery with TD modulation.

We found a main effect of task in reward related areas, especially when we incorporated the parametric modulation of the BOLD-response using the prediction errors (Fig. 2).

### 3.4 Temporal difference-related activity results

**Fig. 3:**
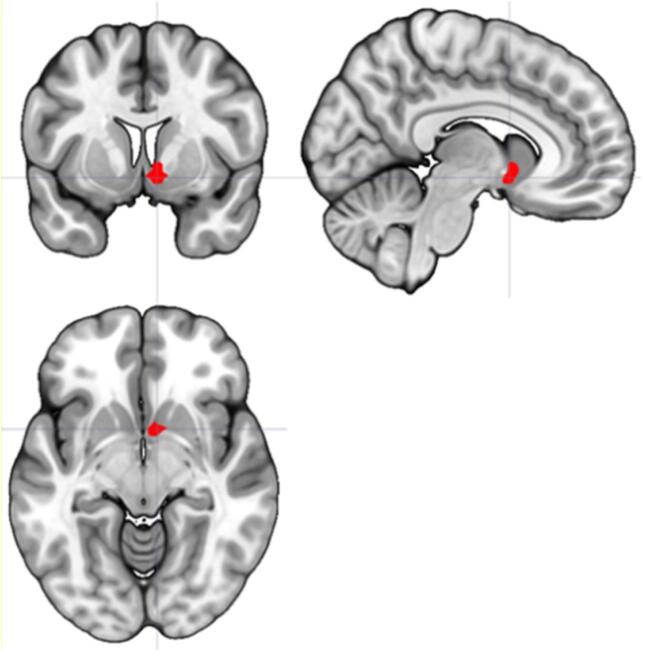
TD-related activity in the ventral striatum (Reward Anticipation + Reward Consumption*TD). MDD patients show decreased VS activity compared to HC (P_FWE,SVC_ = 0.052).

We found a trend towards decreased temporal difference-related activation in the VS in MDD patients compared to HC during reward anticipation and delivery combined (P_FWE,SVC_ = 0.052, Cohen’s d = 0.82, Table 2, Fig. 3). Cohen’s d for the whole right VS (36 voxels) was 0.56.

**Table 2:** Between group TD-related activation ROIs.

Contrast		Location		Voxels	MNI coordinates	*z*	Significance a
RewardAnticip	HC > MDD	VS	R	3	(3, 8, −7)	1.97	0.292
		L	No clusters survived threshold				
		Caudate	R	9	(9, 20, −4)	2.05	0.846
			L	2	(-36, –22, −10)	2.16	0.782
	MDD > HC	VS	R/L	No clusters survived threshold			
		Caudate	R	5	(33, −31, −1)	2.26	0.763
			L	No clusters survived threshold			
RewardConsump*TD	HC > MDD	VS	R	4	(6, 8, −4)	2.31	0.166
			L	5	(-9, 5, −10)	1.85	0.358
		Caudate	R	83	(9, 11, 14)	3.05	0.181
			L	29	(-9, 14, 11)	2.38	0.641
	MDD > HC			No clusters survived threshold			
RewardAnticip + RewConsump*TD	HC > MDD	VS	R	14	(6, 8, −4)	2.83	0.052
			L	4	(-6, 11, −7)	1.97	0.300
		Caudate	R	79	(18, 6, 11)	2.58	0.480
			L	72	(-6, 11, 8)	2.85	0.288
		MDD > HC		No clusters survived threshold			

a FWE peak level corrected + small volume corrected.

### 3.5 Functional connectivity (gPPI) results HC vs MDD

**Fig. 4:**
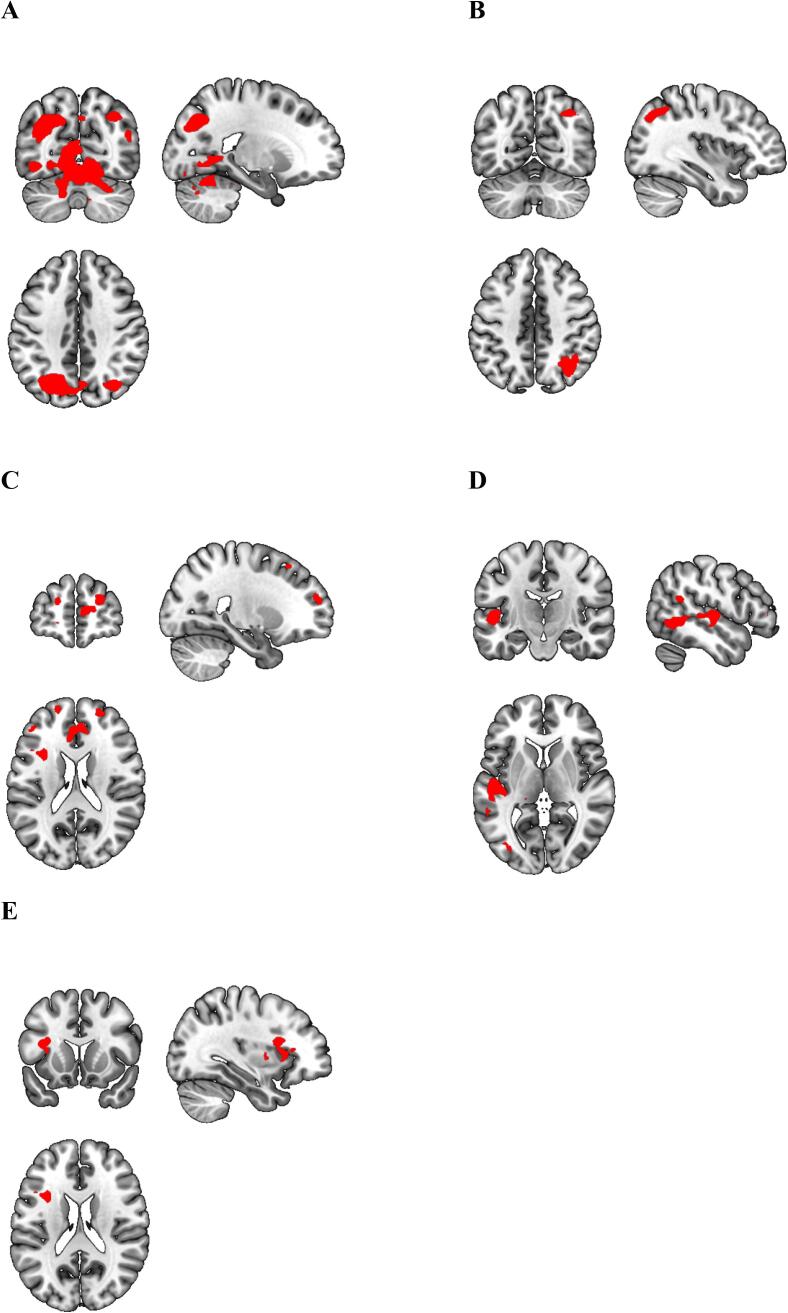
gPPI results VS-seed. During reward anticipation, MDD patients show decreased functional connectivity between the VS and A) Precuneus/superior occipital gyrus/cerebellum *(Z = 4.87, P < 0.001)*, B) Angular gyrus *(Z = 4.37, P = 0.002)*, C) Superior/middle frontal gyrus/medial prefrontal cortex *(Z = 4.21, P < 0.001)*, D) Superior/middle temporal gyrus *(Z = 4.12, P0.001)*, E) Left insula *(Z = 3.96, P = 0.013)*.

**Fig. 5:**
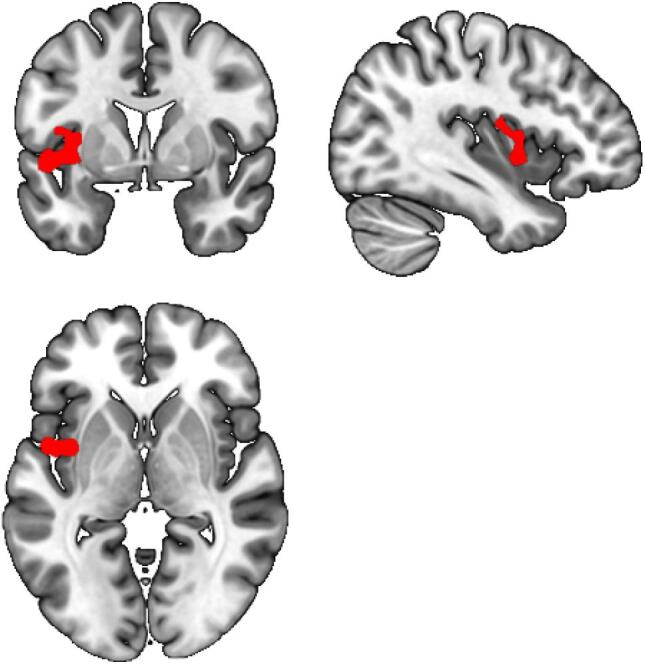
gPPI results VTA-seed. During reward anticipation, MDD patients showed decreased functional connectivity between the VTA and left insula (*Z* = 4.16, *P* < 0.001) compared to HC.

Our gPPI analyses revealed that during reward anticipation, MDD patients exhibited decreased functional connectivity between the VS and precuneus/superior occipital gyrus/cerebellum, angular/middle orbital gyrus, superior/middle frontal gyrus/medial prefrontal cortex (mPFC)/ACC, superior/middle temporal gyrus and left insula compared to HC (Table 3, Fig. 4). Moreover, MDD patients showed decreased functional connectivity between the VTA and left insula compared to HC during reward anticipation (Table 3, Fig. 5). No group differences were found for the DS seed. For all seeds, no group differences were found in functional connectivity during reward delivery.

**Table 3:** Between group gPPI connectivity, HC vs MDD.

seed	Contrast		Location	Voxels	MNI coordinates	*z*	Significance^a^
VS	RewAnticip > NeutralAnticip	HC > MDD	Precuneus/superior occipital gyrus / cerebellum	2659	(-21, −67, 38)	4.87	< 0.001
			Angular/middle orbital gyrus	126	(36, −64, 44)	4.37	0.002
			Superior/middle frontal gyrus / anterior cingulate cortex / medial prefrontal cortex	185	(24, 56, 20)	4.21	<0.001
			Superior/middle temporal gyrus	131	(-51. −16, 5)	4.12	0.001
			Left Insula	83	(–33, 14, 20)	3.96	0.013
		MDD > HC	No clusters survived threshold				
VTA	RewAnticip > NeutralAnticip	HC > MDD	Insula Left	200	(-39, 2, −1)	4.16	< 0.001
		MDD > HC	No clusters survived threshold				
DS	RewAnticip > NeutralAnticip	HC > MDD	No clusters survived threshold				
		MDD > HC	No clusters survived threshold				

a Voxel level p < 0.001 and cluster level FWE corrected at p < 0.017 (0.05/3 for 3 ROIs).

### 3.6 Exploratory analysis: effect of medication

When separating the medication free (MDDmed-) from the antidepressant using MDD patients (MDDmed + ) we found no significant effect of medication status on the temporal difference-related activity in ventral striatum or caudate (data available on request). For the gPPI, we found that compared to HC, MDDmed + and MDDmed- patients showed decreased functional connectivity between the VS and precuneus and areas of the occipital lobe during reward anticipation (Table 4). Moreover, there was a trend in decreased functional connectivity between the VS and mPFC, insula and thalamus (p = 0.025–0.063) in the MDD + patients. This trend was not visible in MDD- patients (all p > 0.187). Furthermore, MDDmed + patients showed decreased functional connectivity between the VTA and left insula compared to HC during reward anticipation (Table 4). No group differences were found in functional connectivity during reward delivery.

**Table 4:** Exploratory analysis, between group gPPI connectivity, HC vs MDDmed + and MDDmed-.

seed	Contrast		Location	Voxels	MNI coordinates	*z*	Significance^a^
VS	RewAnticip > NeutralAnticip	HC > MDDmed^+^	Angular/superior occipital gyrus	296	(36, −58, 47)	5.21	<0.001
			Middle occipital gyrus	371	(-27, −67, 41)	4.90	<0.001
			Precuneus	261	(9, −46, 53)	4.25	<0.001
			Cerebellum	275	(-15, −52, −28)	4.02	<0.001
		HC > MDDmed^-^	Calcarine	817	(-6, −64, 11)	4.06	<0.001
			Precuneus/middle occipital gyrus	92	(-39, −73, 32)	3.95	0.008
		MDD med^+^>HC	No clusters survived threshold				
		MDD med^-^>HC	No clusters survived threshold				
VTA		HC > MDDmed^+^	Insula Left	150	(-51, 2, −1)	4.61	< 0.001
		HC > MDDmed^-^	No clusters survived threshold				
		MDD med^+^>HC	No clusters survived threshold				
		MDD med^-^>HC	No clusters survived threshold				

a FWE cluster level corrected, bonferroni corrected.

### 3.7 Correlation with anhedonia

We found no correlation between temporal difference-related reward activation/connectivity and anhedonia scores, neither when the analysis was corrected for age and gender.

## 4 Discussion

The present study explored temporal difference-related responses of the reward system during a monetary incentive delay task. We demonstrated that parametric modulation of the BOLD-response with prediction errors optimizes monetary incentive task activation. Using the temporal difference, we found decreased temporal difference-related activation in the VS in MDD patients compared to HC during reward anticipation and delivery combined. We found no group differences in temporal difference-related VTA activation. Secondly, we exploratory investigated connectivity between reward circuitry brain areas with gPPI. We revealed that during reward anticipation, MDD patients exhibited an overall decrease in reward circuit connectivity compared to HC. Exploratory analysis separating medication free patients from patients using antidepressants revealed decreased functional connectivity between VTA and left insula in the MDD group that used antidepressants, with an additional trendwise decrease in functional connectivity between the VS and mPFC, insula and thalamus. Of note, all group differences were not related to the reward delivery condition, suggesting that these results are specific to reward anticipation.

The decrease in temporal difference-related activation in the VS is supported by a robust body of evidence showing decreased VS activation in MDD during basic reward processing (Pizzagalli et al., 2009, Ubl et al., 2015). Although our results have to be interpreted with caution, as this effect narrowly missed statistical significance (*P* = 0.052 FWE/SVC-corrected), this finding is bolstered by the fact that it also replicates previous results specifically investigating temporal difference-related VS activation (Kumar et al., 2008). No differences in reaction times were observed between groups, indicating that fMRI findings were not confounded by differences between groups in task difficulties. A similar lack of group differences on behavioral responses has been reported before (Knutson et al., 2008, Pizzagalli et al., 2009, Ubl et al., 2015).

Impaired reward functioning as a construct relevant for depression is further corroborated by our gPPI findings of decreased functional connectivity between the reward system and several other brain areas including the insula. It should be noted that the good fit of our gPPI analyses might be a result of relatively poor fit of the basic reward anticipation model. The insula has been linked to anticipating future rewards (Tanaka et al., 2004) and delayed gratification (Wittmann et al., 2007). Moreover, a recent *meta*-analysis of 42 studies has demonstrated functional connectivity between the VS and the insula (Cauda et al., 2011). This connectivity is critical in detecting salient external stimuli and adjust behavior to these incentives (Cho et al., 2013). Our observation of decreased VS-insula connectivity during anticipation of rewards in MDD suggests that MDD patients have difficulties in integrating salient information into motivational processes to shape behavior. Besides this involvement, insula activity also appears during PE encoding of reward (Haruno and Kawato, 2006, Jones et al., 2011), suggesting encoding of a salience PE (Gu et al., 2016, Metereau and Dreher, 2013). The decreased VTA-insula functional connectivity in MDD suggests an impairment in encoding these salience PEs.

We also found decreased functional connectivity between the VS and the mPFC and superior/middle frontal gyrus during reward anticipation in MDD patients. Animal studies provide fundamental evidence that the mPFC is part of the reward system and is involved in reward seeking and reward effort (Tzschentke, 2000). The mPFC receives dopaminergic projections from the VTA and sends glutamatergic projections back to the VTA and VS These functional interactions have been suggested to strongly modulate the mesocorticolimbic dopamine circuit (Tzschentke and Schmidt, 2000) and have been suggested to be specifically related to reward anticipation (Balleine et al., 2007, Knutson et al., 2001, Wittmann et al., 2007). Animal studies report that inactivation of the mPFC reduces the firing rate of VS neurons in response to reward-predictive cues (Ishikawa et al., 2008). Disrupted functional connectivity from the VS to the mPFC during anticipation could hamper activation of the mPFC, which in turn may decrease alter the feedback projections to the VTA and VS resulting in mesolimbic reward circuitry abnormalities. These current results substantiate the notion that dysfunctions in fronto-striatal activity during reward anticipation are an important marker of MDD (Zhang et al., 2013).

Besides their role in the reward circuitry, the ACC/mPFC are, together with the precuneus, important areas of the default mode network (DMN). In healthy controls, functional connectivity has been reported between the VS and DMN regions including the precuneus and mPFC (Di Martino et al., 2008). A previous study in depressed individuals found that compared with controls, depressed subjects showed decreased connectivity between the precuneus/PCC and the striatum (Bluhm et al., 2009), which is in line with the current results. The DMN has been found to support internal mental activity and is also critical for self-relevance and self-referential processing (Raichle, 2015). It is possible that decreased VS-DMN connectivity causes an impairment in assigning salience to external and internal stimuli, potentially leading to aberrant salience.

Our analyses also revealed decreased functional connectivity between the VS and the cerebellum during reward anticipation in MDD patients. Interestingly several recent studies have pointed to the important connection between the cerebellum and striatum during the anticipation of reward. Animal studies provided insight in the role of cerebellum in the encoding of reward which may serve to be integrated in goal directed behavior (Wagner et al., 2017) and there are also direct projections from the cerebellum to reward centers in the brain (Carta et al., 2019). In humans there is also accumulating evidence that the cerebellum is active during reward anticipation phase of the monetary incentive delay tasks (Wilson et al., 2018), while there are reciprocal connections between cerebellum and striatum (for a review see Milardi et al., 2019). It is possible that decreased VS-cerebellar connectivity impairs the flexible integration of reward and cognitive resources in the context of motivated behavior in depression. Schutter (2016) has already pointed to the important role of the cerebellum in depression, via its contributing role to reward-related predictive coding in the service of homeostasis.

When separating the medication free patients from the patients using antidepressants, we found that the decreased connectivity patterns were mainly apparent in the MDD group that used antidepressants. Given the association between antidepressant use and diminished neural responses of the reward system (McCabe et al., 2010), and the suggestion that SSRI treatment blunts dopaminergic activity, explaining symptoms such as anhedonia and affective blunting (Goodwin et al., 2017), it can be argued that reward related connectivity may be affected by antidepressant treatment, however, this remains entirely speculative.

No differences between groups were observed in temporal difference-related activity during reward delivery. This finding is in line with studies by Stoy and colleagues (Stoy et al., 2012) and Ubl and colleagues (Ubl et al., 2015), who also report depression related dysfunctions during reward anticipation but not during the receipt of reward. Given that other studies report decreased fronto-cingulate-striatal activation during the reward delivery phase (Forbes et al., 2009, Knutson et al., 2008, Pizzagalli et al., 2009), and considering the modest sample size of our study, our null findings should be interpreted with caution. Future studies should reveal the extent of dysfunctions during reward delivery in MDD.

The present study did not identify a correlation between brain activation/connectivity of the reward system and hedonic capacity. This lack of an association is in contrast to other papers (Chase et al., 2010, Ubl et al., 2015). However, differences in task paradigms and anhedonia questionnaires might account for these differences. E.g., Chase and colleagues (Chase et al., 2010) used a probabilistic selection task and Ubl and colleagues (Ubl et al., 2015) employed a modified version of the MID task we used. In both studies hedonic capacity was measured with the Snaith Hamilton Pleasure Scale (SHAPS), while we assessed anhedonia with the BDI anhedonia subscore, which resulted in a narrow range of anhedonia scores. The SHAPS embodies a more extensive measurement of consummatory anhedonia which may have been more sensitive in mapping anhedonia levels.

### 4.1 Strengths and limitations

The current design enabled us to explore functional connectivity alterations in the reward circuitry, which is a novel feature compared to measuring altered activity of reward related brain areas during reward processing, as supported by previous work (Admon et al., 2015a, Pizzagalli et al., 2009, Smoski et al., 2009, Zhang et al., 2013). Furthermore, this study is novel in modeling temporal difference signals in a MID task which might give a more accurate representation of reward-related brain activity and connectivity. Nevertheless, potential limitations exist. First, no temporal difference-related VTA task activity was found. The nature of the task used in this study may account for the absence of temporal difference-related activity in the VTA. Traditionally, the MID task has been designed to investigate changes in neural activity in response to basic processing of reward. Activation in the VTA, elicited from firing of dopaminergic neurons during reward-related learning, is most likely best reflected by a classical conditioning paradigm, for example used by Kumar et al. (2008). Second, in our approach we restricted our analyses to a previously validated TD-model of reward anticipation (Staudinger et al., 2009), however more recent drift diffusion models like a complete serial compound (SCS) TD model might show better fit of the data (Luzardo, 2018). Third, ten out of twenty-four MDD patients were receiving antidepressants at time of scanning. Splitting up the patient group into two group in order to rule out any medication effects on the results, showed detrimental effects of antidepressants on reward processing. However, this resulted in small sample sizes per subgroup. Interpretation of these results should therefore be done with caution until they can be replicated in larger samples. Third, the overall sample size of our study with 48 participants in total, and 24 per condition, is generally at the lower end of the spectrum for a neuroimaging study and therefor runs the risk of type 2 error, i.e. false negative findings. Because, however, our study has a clear theoretical framework, clear hypothesis, and we found robust task activations (Fig. 2), we are confident in the findings of our study.

## 5 Conclusion

The present study showed that MDD is characterized rather by alterations in reward circuit connectivity than isolated activation impairments in brain areas underlying the reward-system. These findings represent an important extension of the existing literature since improved understanding of neural pathways underlying depression-related reward dysfunctions, may help currently unmet diagnostic and therapeutic efforts. The finding that antidepressants might decrease connectivity in the reward-system requires future research with primary interest in the effects of antidepressants in larger samples.

## Declaration of Competing Interest

The authors declare that they have no known competing financial interests or personal relationships that could have appeared to influence the work reported in this paper.

## Data Availability

Data will be made available on request.
